# Patterns of Complementary and Alternative Medicine Use in Saudi Arabian Patients With Inflammatory Bowel Disease: A Cross-Sectional Study

**DOI:** 10.7759/cureus.9687

**Published:** 2020-08-12

**Authors:** Albaraa Altunisi, Mahmoud Mosli, Mazen Banweer, Yousif Qari, Faris O Arif, Omar I Saadah

**Affiliations:** 1 Internal Medicine, King Abdulaziz University Hospital, Jeddah, SAU; 2 Gastroenterology, King Abdulaziz University Hospital, Jeddah, SAU; 3 Pediatrics, King Abdulaziz University Hospital, Jeddah, SAU; 4 Pediatric Gastroenterology, King Abdulaziz University Hospital, Jeddah, SAU

**Keywords:** inflammatory bowel disease, chron's, ulcerative colitis, ibd, complementary and alternative medicine, cam

## Abstract

Background

Inflammatory bowel disease (IBD) concerns a group of chronic autoimmune diseases that results in uncontrolled inflammation of the gastrointestinal (GI) tract, which can lead to long-term complications. Conventional treatments for IBD usually target inflammation and include biologics and immunosuppressants, which have been associated with significant adverse effects. Also, non-response to biologics has been reported in up to 50% of patients. Hence, patients sometimes turn to unconventional methods of treatment, such as complementary and alternative medicine (CAM). In this study, we examine patterns of CAM use in Saudi patients diagnosed with IBD.

Materials and methods

We conducted a cross-sectional study of patients with IBD who were seen at the outpatient gastroenterology clinic between January 2018 and December 2019. Adult patients diagnosed with ulcerative colitis (UC) or Crohn’s disease (CD) were identified and surveyed. Clinical, laboratory, endoscopic, radiologic, and histologic data were collected. The patients completed a detailed questionnaire focusing on CAM use. Descriptive statics, quantitative variables, means, standard deviations (SDs), and minimum and maximum values or medians with interquartile ranges were used where appropriate; for qualitative variables, we reported frequencies. The prevalence of CAM use was calculated using standard prevalence formulae. Logistic regression analysis was performed to identify predictors for CAM use. A p-value of <0.05 was set as statistically significant.

Results

A total of 71 IBD patients were surveyed, of which 75% had CD. Severe symptoms were reported by 54% of patients, and 41% were receiving biological treatment; 90% of this cohort reported using some form of CAM, of which 78% used them within the past year, and 52% reported CAM treatment having a positive effect. Of note, 63% of patients reported using CAM therapy without the knowledge of their treating physician. The most common source of advice regarding the use of CAM medicine was relatives (66%), and the most common forms of CAM used were honey (62%), Zamzam water (54%), and physical activity (32%). Binary logistic regression analysis identified watery diarrhea (OR=5.7, 95% CI=1.0-31, P=0.04) and azathioprine (OR=18.1, 95% CI=1.3-255, P=0.03) as predictors of CAM use.

Conclusions

CAM use is very common in Saudi patients with IBD. The majority of patients seem to turn to CAM if their symptoms are severe. They generally appear to be influenced by culture, the Internet, local society, and family tradition in their decision to use CAM, rather than expert advice from their treating physician.

## Introduction

Inflammatory bowel disease (IBD) concerns a group of chronic inflammatory conditions of indefinite etiology that comprises two main disorders: Crohn’s disease (CD), which can affect any part of the gastrointestinal (GI) tract, from mouth to anus; and ulcerative colitis (UC), which primarily involves the mucosa of the colon. IBD is characterized by relapsing-remitting symptoms that reflect progressive bowel damage, which eventually leads to the development of complications, resulting in impaired quality of life [1]. Conventional treatments for IBD include anti-inflammatory medications such as corticosteroids, 5-aminosalicylic acid derivatives, immunomodulators such as azathioprine, and biological therapies [2]. The main function of all available treatment options is to induce and maintain symptom-free remission and mucosal healing [3]. The risk of requiring surgery due to complications of IBD or treatment failure currently ranges between 20-80% within 10 years of diagnosis [4]. In response to these contingencies, patients with IBD often seek unconventional modalities of treatment, such as complementary and alternative medicine (CAM) [5]. CAM comprises various medical procedures, healthcare practices, and products that are usually unrelated to conventional medical practice [6]. CAM involves a variety of forms, including spiritual therapies (healing, prayer, and meditation), herbal medicine, Chinese medicine, homeopathic medicine, Hindu remedies, and biological products (probiotics, vitamins, and minerals) [7-9].

The use of CAM seems to be relatively common in many Western and non-Western cultures, including Saudi Arabia, owing to the overall perception held by many patients that it is less toxic and less hazardous than many conventional medical treatments [10]. In this study, we surveyed Saudi Arabian patients who suffer from IBD about their experiences with CAM in the treatment of their diseases.

## Materials and methods

We conducted a cross-sectional study of all adult patients (aged 18-85 years) with confirmed IBD who followed up at the outpatient gastroenterology clinic, between January 2018 and December 2019. Data on demographics, clinical disease characteristics, treatments, and comorbidities were collected. A pre-designed questionnaire was used to record information on participants’ CAM usage. Our questionnaire consisted of questions regarding the following aspects: personal data such as age, gender, marital status, and educational level; the history of IBD including onset, symptoms, causes, severity, and complications; details relating to the use of CAM for the treatment of IBD, including onset, benefits, frequency, and side effects (see Appendices).

The primary outcome measure of the study was to estimate the prevalence of CAM usage in patients with IBD. The secondary outcome was to evaluate patient response to CAM usage, such as whether there were any side effects, and to identify associations between patient characteristics and patterns of CAM usage.

Data were analyzed using SPSS Statistics Version 22 (IBM, Armonk, NY). Baseline descriptions were calculated: frequencies and percentages were generated for categorical variables, while means and standard deviations (SDs) were determined for quantitative variables. The prevalence rate was quantified and presented as percentages. Chi-square and Fisher’s exact tests were used to test the association between categorical variables. Binary logistic regression analysis was performed to look for predictors of CAM use. A p-value of <0.05 was set as statistically significant.

## Results

A total of 71 IBD patients were surveyed, of whom 53 (75%) had CD and 18 (25.4%) had UC. The main sociodemographic and clinical features are shown in Table 1. Ten patients (14%) were younger than 20 years of age, 53 patients (75%) were between 20-40 years, and eight patients (11%) were older than 40 years. Forty-six patients (64.8%) had a disease duration of equal to or less than six years, and 25 patients (35.2%) had a disease duration of more than six years. Females constituted 45% of patients (n=32); 42% of the whole cohort (n=30) were married, 54% (n=38) held a university degree, and 46.5% (n=33) had a monthly income of less than 5,000 Saudi riyals. The main presenting symptoms in patients were as follows: abdominal pain (90%), diarrhea (65%), nausea and vomiting (61%), and rectal bleeding (54%). Severe symptoms were reported by 54% of patients, and 39% faced constraints in their daily activities due to disease severity.

**Table 1:** Clinical and demographic characteristics of the study population TNF: tumor necrosis factor

		Ulcerative colitis, n (%); n=18	Crohn’s disease, n (%); n=53	Total, n (%); n=71
Sociodemographic characteristics				
Age group (years)	<20	3 (16.7%)	7 (13.2%)	10 (14.1%)
	20–40	10 (55.6%)	43 (91.1%)	53 (74.6%)
	>40	5 (27.8%)	3 (5.7%)	8 (11.3%)
Gender	Female	10 (55.6%)	22 (41.5%)	32 (45.1%)
	Male	8 (44.4%)	31 (58.5%)	39 (54.9%)
Marital status	Married	10 (55.6%)	20 (37.7%)	30 (42.3%)
	Unmarried	8 (44.4%)	33 (62.3%)	41 (57.7%)
Smoking		2 (11.1%)	12 (22.6%)	14 (19.7%)
Educational level	Elementary school	2 (11.1%)	2 (3.8%)	4 (5.6%)
	High school and diploma	6 (33.3%)	23 (43.4%)	29 (40.9%)
	University degree	10 (55.6%)	28 (52.8%)	38 (53.5%)
Household income per month (Saudi riyal)	<5,000	6 (33.3%)	27 (50.9%)	33 (46.5%)
	5,000–10,000	6 (33.3%)	21 (39.6%)	27 (38%)
	>10,000	6 (33.3%)	5 (9.4%)	11 (15.5%)
Residential area	Urban	17 (94.4%)	52 (98.1%)	69 (97.2%)
	Rural	1 (5.6%)	1 (1.9%)	2 (2.8%)
Clinical features				
Disease duration (years)	≤6	12 (66.7%)	34 (64.2%)	46 (64.8%)
	>6	6 (33.3%)	19 (35.8%)	25 (35.2%)
Symptoms	Abdominal pain	15 (83.3%)	49 (92.5%)	64 (90.1%)
	Watery diarrhea	14 (77.8%)	32 (60.4%)	46 (64.8%)
	Nausea/vomiting	9 (50%)	34 (64.2%)	43 (60.6%)
	Rectal bleeding	15 (83.3%)	23 (44.2%)	38 (54.3%)
Medications	Corticosteroids	4 (22.2%)	10 (18.9%)	14 (19.7%)
	Mesalamine	6 (33.3%)	11 (20.8%)	17 (23.9%)
	Azathioprine	2 (11.1%)	18 (34%)	20 (28.2%)
	Anti-TNF therapy	4 (22.2%)	25 (47.2%)	29 (40.8%)

**Figure 1:**
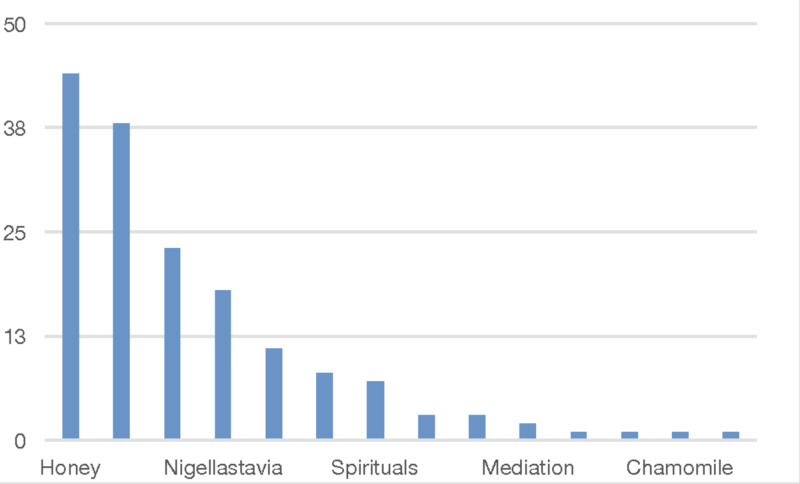
Complementary medicine therapies used by patients with IBD IBD: inflammatory bowel disease

The use of at least one form of CAM therapy, such as dietary, herbal, spiritual, or physical, was reported by 64 IBD patients (90%) in this cohort. Eliminating the physical and spiritual therapies resulted in a prevalence rate of CAM use of 77.5% (n=55). The most commonly used CAM therapies as reported by patients were as follows: honey (n=44, 62%); Zamzam water (n=38, 54%); motion and physical activity (n=23, 32%); Nigella sativa (black seeds) (n=18, 25%); ginger (n=11, 15.5%); and turmeric (curcumin) (n=8, 11%) (Figure 1). Patients who had previously used CAM reported using it at a frequency of two to five times (19.7%), 6-10 times (26.8%), or >10 times (36.6%). The majority of patients using CAM (n=45, 63%) said they did not disclose CAM use to their treating physician.

Thirty-seven patients (52%) experienced a positive effect with CAM use and eight (11%) reported having side effects after use. Their knowledge about CAM use was obtained through relatives (n=43, 66%), friends (n=10, 15%), the Internet (n=8, 11%), caregivers (n=7, 10%), or religious advisors (n=3, 4%).

Bivariate analysis of various sociodemographic and clinical variables and the use of CAM therapy, excluding physical and spiritual form (Table 2), showed associations between male gender and the use of black seeds (P=0.001), smoking and turmeric (P=0.04), smoking and ginger (P=0.005), low education level and honey (P=0.03), low education level and black seeds (P=0.02), abdominal pain and turmeric (P=0.03), rectal bleeding and Zamzam water (P=0.03), and Zamzam water and azathioprine therapy (P=0.008).

**Table 2:** Bivariate analysis of sociodemographic and clinical variables and the use of dietary and herbal CAM therapies CAM: complementary and alternative medicine; IBD: inflammatory bowel disease; UC: ulcerative colitis; CD: Crohn’s disease; TNF: tumor necrosis factor

		Honey			Zamzam water			Black seeds			Curcumin			Ginger		
Characteristics		Yes, n (%)	No, n (%)	P-value	Yes, n (%)	No, n (%)	P-value	Yes, n (%)	No, n (%)	P-value	Yes, n (%)	No, n (%)	P-value	Yes, n (%)	No, n (%)	P-value
Gender	Male	27	12	0.22	24	15	0.16	16	23	0.001	5	34	0.72			0.72
	Female	17	15		14	18		2	30		3	29				
Age group (years)	≤40	37 (84.1)	26 (96.3)	0.14	34 (89.5)	29 (87.9)	1.0	16 (88.9)	47 (88.7)	1.0	7 (87.5)	56 (88.9)	1.0	8 (72.7)	55 (91.7)	0.10
	>40	7 (15.9)	1 (3.7)		4 (10.5)	4 (12.1)		2 (11.1)	6 (11.3)		1 (12.5)	7 (11.1)		3 (27.3)	5 (8.3)	
Marital status	Married	21 (47.7)	9 (33.3)	0.32	19 (50)	11 (33.3)	0.23	8 (44.4)	22 (41.5)	1.0	3 (37.5)	27 (42.9)	1.0	7 (63.6)	23 (38.3)	0.18
	Unmarried	23 (52.3)	18 (66.7)		19 (50)	22 (66.7)		10 (55.6)	31 (58.5)		5 (62.5)	36 (57.1)		4 (36.4)	37 (61.7)	
Smoking	Yes	11 (25)	3 (11.1)	0.22	9 (23.7)	5 (15.2)	0.28	6 (33.3)	8 (15.1)	0.09	4 (50)	10 (15.9)	0.04	6 (54.5)	8 (13.3)	0.005
	No	33 (75)	24 (88.9)		29 (76.3)	28 (84.8)		12 (66.7)	45 (84.9)		4 (50)	53 (84.1)		5 (45.5)	52 (86.7)	
Education	Before university	25 (56.8)	8 (29.6)	0.03	21 (55.3)	12 (36.4)	0.15	13 (72.2)	20 (37.7)	0.02	5 (62.5)	28 (44.4)	0.46	6 (54.5)	27 (45)	0.74
	University	19 (43.2)	19 (70.4)		17 (44.7)	21 (63.6)		5 (27.8)	33 (62.3)		3 (37.5)	35 (55.6)		5 (45.5)	33 (55)	
Monthly Income (Saudi riyal)	<1,000	35 (79.5)	25 (92.6)	0.19	32 (84.2)	28 (84.8)	1.0	15 (83.3)	45 (84.9)	1.0	7 (87.5)	53 (84.1)	1.0	8 (72.7)	52 (86.7)	0.36
	>1,000	9 (20.5)	2 (7.4)		6 (15.8)	5 (15.2)		3 (16.7)	8 (15.1)		1 (12.5)	10 (15.9)		3 (27.3)	8 (13.3)	
Residential area	Urban	42 (95.5)	27 (39.1)	0.52	37 (97.4)	32 (97)	1.0	16 (88.9)	53 (100)	0.06	7 (87.5)	62 (98.4)	0.21	10 (90.9)	59 (98.3)	0.29
	Rural	2 (4.5)	0 (0.0)		1 (2.6)	1 (3.0)		2 (11.1)	0 (0.0)		1 (12.5)	1 (1.6)		1 (9.1)	1 (1.7)	
IBD type	UC	12 (27.3)	6 (22.2)	0.78	9 (23.7)	9 (27.3)	0.79	5 (27.8)	13 (24.5)	0.76	0 (0.0)	18 (28.6)	0.11	5 (45.5)	13 (21.7)	0.13
	CD	32 (72.7)	21 (77.8)		29 (76.3)	24 (72.7)		13 (72.2)	40 (75.5)		8 (100)	45 (71.4)		6 (54.5)	47 (78.3)	
Abdominal pain	Yes	41 (93.2)	23 (85.2)	0.42	36 (94.7)	28 (84.8)	0.24	16 (88.9)	48 (90.6)	1.0	5 (62.5)	59 (93.7)	0.03	11 (100)	53 (88.3)	0.59
	No	3 (6.8)	4 (14.8)		2 (5.3)	5 (15.2)		2 (11.1)	5 (9.4)		3 (37.5)	4 (6.3)		0 (0.0)	7 (11.7)	
Watery diarrhea	Yes	32 (72.7)	14 (51.9)	0.12	26 (68.4)	20 (60.6)	0.62	14 (77.8)	32 (60.4)	0.26	7 (87.5)	39 (61.9)	0.25	9 (81.8)	37 (61.7)	0.17
	No	12 (27.3)	13 (48.1)		12 (31.6)	13 (39.4)		4 (22.2)	21 (39.6)		1 (12.5)	24 (38.1)		2 (18.2)	23 (38.3)	
Nausea/vomiting	Yes	30 (68.2)	13 (48.1)	0..13	26 (68.4)	17 (51.5)	0.22	13 (72.2)	30 (56.6)	0.28	5 (62.5)	38 (60.3)	1.0	7 (63.6)	36 (60)	1.0
	No	14 (31.8)	14 (51.9)		12 (31.6)	16 (48.5)		5 (27.8)	23 (43.4)		3 (37.5)	25 (39.7)		4 (36.4)	24 (40)	
Rectal bleeding	Yes	27 (61.4)	11 (42.3)	0.13	25 (67.6)	13 (39.4)	0.03	12 (66.7)	26 (50)	0.28	4 (50)	34 (54.8)	1.0	9 (81.8)	29 (49.2)	0.05
	No	17 (38.6)	15 (57.7)		12 (32.4)	20 (60.6)		6 (33.3)	26 (50)		4 (50)	28 (45.2)		2 (18.2)	30 (50.8)	
Corticosteroids	Yes	12 (27.3)	2 (7.4)	0.06	9 (23.7)	5 (15.2)	0.55	5 (27.8)	9 (17)	0.32	2 (25)	12 (19)	0.65	2 (18.2)	12 (20)	1.0
	No	32 (72.7)	25 (92.6)		29 (76.3)	28 (84.8)		13 (72.2)	44 (83)		6 (75)	51 (81)		9 (81.8)	48 (80)	
Azathioprine	Yes	15 (34.1)	5 (18.5)	0.18	16 (42.1)	4 (12.1)	0.008	5 (27.8)	15 (28.3)	1.0	2 (25)	18 (28.6)	1.0	2 (18.2)	18 (30)	0.72
	No	29 (65.9)	22 (81.5)		22 (57.9)	29 (87.9)		13 (72.2)	38 (71.7)		6 (75)	45 (71.4)		9 (81.8)	42 (70)	
Anti-TNF-alpha	Yes	16 (36.4)	13 (48.1)	0.46	15 (39.5)	14 (42.4)	0.81	6 (33.3)	23 (43.4)	0.58	3 (37.5)	26 (41.3)	1.0	5 (45.5)	24 (40)	0.75
	No	28 (63.6)	14 (51.9)		23 (60.5)	19 (57.6)		12 (66.7)	30 (56.6)		5 (62.5)	37 (58.7)		6 (54.5)	36 (60)	

Binary logistic regression analysis using CAM therapy as the dependent variable identified the presence of watery diarrhea (OR=5.7, 95% CI=1.0-31, P=0.04) and the use of azathioprine (OR=18.1, 95% CI=1.3-255, P=0.03) as predictors of CAM use (Table 3).

**Table 3:** Binary logistic regression analysis of sociodemographic and clinical variables as predictors of the use of CAM therapy in IBD patients *P-value statistically significant CAM: complementary and alternative medicine; IBD: inflammatory bowel disease; TNF: tumor necrosis factor

Variable	Odds ratio	95% confidence interval		P-value
		Lower	Upper	
Gender	2.1	0.49	8.9	0.32
Marital status	1.6	0.29	8.5	0.58
Education	1.5	0.34	6.8	0.59
Monthly income	4.2	0.39	44	0.23
Smoking	1.9	0.21	17.7	0.57
IBD types	0.92	0.14	6	0.93
Abdominal pain	0.56	0.05	6.5	0.64
Watery diarrhea	5.7	1	31	0.046*
Nausea and vomiting	1.1	0.19	5.6	0.95
Rectal bleeding	1.8	0.36	8.9	0.48
Corticosteroids	2.5	0.33	18.9	0.38
Azathioprine	18.1	1.3	255	0.03*
Anti-TNF-alpha therapy	2	0.37	11.4	0.41

## Discussion

Patients diagnosed with IBD exhibit symptoms that may interfere with their ability to function and maintain a good quality of life [11]. Available medical treatments, ranging from corticosteroids to biological therapies, carry a wide range of efficacy and safety profiles [2,12]. Anti-tumor necrosis factor (TNF) agents are considered one of the most effective classes of treatments for IBD, although 30% of patients treated with anti-TNF agents do not respond to the treatment (termed as primary non-responders), and another 30% of responders tend to lose response with time (termed secondary non-responders) [13]. In addition, patients treated with biologics may develop adverse events that range from simple recurrent sinusitis to severe pneumonia [10,14,15]. As such, patients are usually searching for further effective and safe treatment options and, hence, often turn to CAM. Previous studies have reported a wide range of CAM agents used for IBD [16,17]. A recent study that surveyed 1,286 Austrian patients with IBD reported that 50.7% used CAM at some point during the disease course [18]. A recent systematic review, published in 2017 by Alrowais et al., estimated a prevalence rate of CAM usage of 90% in Saudi Arabian patients [10]. Our results show a corresponding prevalence rate of CAM usage in IBD patients in Saudi Arabia (90%).

Previous studies have noted that female gender and higher degrees of education, as well as longer duration of the disease, were associated with CAM usage [19,20]. However, our study has shown that IBD patients of male gender and lower levels of education were associated with the use of black seeds and honey, respectively. Patients treated with corticosteroids and anti-TNF agents also reported frequent use of CAM, and the most frequently used CAM therapies by this cohort were prayer, meditation, herbal remedies, homeopathy, and mind-body techniques [18]. We did not find that treatment with corticosteroids or anti-TNF-alpha were associated with CAM; however, our results suggest a significant association between treatment with azathioprine and the use of Zamzam water. Another study from Chile, which included 200 IBD patients, reported a 55% prevalence rate of CAM use and that 49% of patients were advised to do so by their friends and relatives [21]. The most commonly used CAM in their cohort was herbal medicine (55%). In our study, honey and Zamzam water appeared to be the most favored CAM choice among IBD patients. Some studies have suggested CAM as a potentially effective therapy for IBD. A recent Cochrane systematic review evaluated non-traditional therapies for IBD, concluding that dietary manipulation, cannabis, and naltrexone were not effective in treating IBD [22]. However, there is some evidence from small and randomized controlled trials that support the efficacy of turmeric as an induction and maintenance adjunctive treatment in UC [23-25]. Turmeric use was reported by 11% of our cohort and was associated with the presence of abdominal pain and lack of smoking.

A number of particular patient characteristics are commonly associated with CAM use. A study by Koning et al., which included 1,370 patients with IBD, revealed that female gender, young age, high education level, high income, being a vegetarian, and being part of the middle social class at birth were independent variables associated with oral CAM use in IBD patients [26]. However, in our study, watery diarrhea and treatment with azathioprine were identified as predictors for CAM use. Previous reports have also highlighted that patients with IBD who turn to CAM are less likely to discuss CAM with their treating physician, possibly due to fear of judgment or interference with their wishes to pursue this path [27-29]. Similarly, in our study cohort, the majority of patients using CAM (63%) did not disclose CAM use to the treating physician, consistent with previous reports.

We recognize that the results presented in this study are limited by the cross-sectional design, the small sample size, and the single-center data source. Hence, we encourage investigators of CAM usage in IBD patients to conduct larger, multicenter prospective studies.

## Conclusions

Our findings reveal that CAM use is very common in Saudi patients with IBD. The majority of patients seem to turn to CAM use when their symptoms are severe, and appear to be influenced by family advice, culture, tradition, Internet forums, and friends, rather than advice given by their treating physician.

## Appendices

Questionnaire on complementary and alternative medical treatment (CAM)

Thank you for taking the time to be part of this investigation, the purpose of which is to examine the use of complementary and alternative medical treatment to highlight the possible positive and negative effects of the treatment and to examine it in the context of public health and quality of life. Even if you have not used any of these treatments, your answers to the questions in the survey are important. Answer the questions by ticking the boxes next to the answer options that you deem fit, and write on the lines for other response and follow the instructions. (You can choose more than one answer sometimes as appropriate).

Complementary and alternative medical treatments are usually abbreviated as CAM. The following four pages contain a list of the different CAM treatments. 

General information

1. Age: 20> 20-40 40-60 60<

2. Gender:  Man Woman

3. Marital Status: Married Single Divorced/separated Widow/widower  

4. What is your education level? Basic school/public school Secondary/qualified vocational school Senior high school College/University ...........…    

5. What kind of area do you live in?  Urban Rural  other  

6. What is your monthly income?  

<5,000 5,000-10,000 >10,000

Lifestyle-related questions 

7. What kind of food do you eat?

 Normal diet  Vegetarian diet (no animal products)  Other diets

8. Do you smoke?  Yes   No

8a. Enter the average number of cigarettes on a daily basis...........…  

9. Do you drink alcohol?  Yes   No

Medical questions

10. Do you have any of the following inflammatory diseases?

 Ulcerative colitis (UC)

 Crohn's disease (CD)

11. When were you diagnosed? >3 years  3-6 years <6 years

12. Do you have any symptoms of your disease at the moment?

Nausea & vomiting  Abdominal pain Diarrhea Bloody diarrhea  Weight loss Fever

13. What is the severity of the disease?

No symptoms Mild Moderate Severe

14. Does the disease affect your daily activities?

Not at all Mildly Moderately Severely

**Table 4:** Questionnaire

Complementary options medical treatment	Have you used this treatment in the last year?	How many times have use this treatment in the last year?	Have you experienced any positive effects of this treatment?	Have you experienced any side-effects or undesirable effects of this treatment?	Does your regular doctor know that you are using this treatment?
Spirituality/religion	q Yes q No	q 1 q 2- 5 q 6-10 q >10	q Yes q No	q Yes q No	q Yes q No
Motion/physical activity	q Yes q No	q 1 q 2- 5 q 6-10 q >10	q Yes q No	q Yes q No	q Yes q No
Nigella sativa	q Yes q No	q 1 q 2- 5 q 6-10 q >10	q Yes q No	q Yes q No	q Yes q No
Zamzam water	q Yes q No	q 1 q 2- 5 q 6-10 q >10	q Yes q No	q Yes q No	q Yes q No
Complementary options medical treatment	Have you used this treatment in the last year?	How many times have use this treatment in the last year?	Have you experienced any positive effects of this treatment?	Have you experienced any side-effects or undesirable effects of this treatment?	Does your regular doctor know that you are using this treatment?
Natural resources or natural products, if YES specify what kind? ______________ ________________________________ Lemon, curcumin, ginger ________________________________	q Yes q No	q 1 q 2- 5 q 6-10 q >10	q Yes q No	q Yes q No	q Yes q No
Honey	q Yes q No	q 1 q 2- 5 q 6-10 q >10	q Yes q No	q Yes q No	q Yes q No
yoga	q Yes q No	q 1 q 2- 5 q 6-10 q >10	q Yes q No	q Yes q No	q Yes q No
Meditation	q Yes q No	q 1 q 2- 5 q 6-10 q >10	q Yes q No	q Yes q No	q Yes q No
Psychotherapy	q Yes q No	q 1 q 2- 5 q 6-10 q >10	q Yes q No	q Yes q No	q Yes q No
Vitamins	q Yes q No	q 1 q 2- 5 q 6-10 q >10	q Yes q No	q Yes q No	q Yes q No
Probiotics	q Yes q No	q 1 q 2- 5 q 6-10 q >10	q Yes q No	q Yes q No	q Yes q No
Additional information: ................................................	q Yes q No	q 1 q 2- 5 q 6-10 q >10	q Yes q No	q Yes q No	q Yes q No

15. What positive effect have you experienced from your CAM treatment according to the list on the previous pages?

16. What adverse reactions (unwanted effects) have you experienced from your CAM treatment according to the list on the previous pages?                                                                                                                                                         

17. If you used the CAM treatment, how did you get information about the CAM treatment?

q Through a friend q Internet q TV q Health food business q operators of CAM q Relative q Consultation from the healthcare provider

q Other                               

18. If you have ever used you of any kind of CAM treatment, indicate the way in which you have used it?

q Together/simultaneously with your normal medical treatment

q Instead of your regular medical treatment

19. Do you take any medicine for your inflammatory disease?

q Yes q No

19a. Which medicines are you using?                               

20. If you have any medical treatment for your inflammatory disease, have you experienced any side effects (unwanted effects) of the medical treatment?

q Yes q No

20a. What are the medications? 

20b. What are the side effects?                          

21. To what extent do you perceive complementary and alternative medical treatment has helped you to manage your life situation? (select the corresponding figure)

In a very high degree            not at all

1          2         3         4         5  

22. To what extent do you perceive complementary and alternative medical treatment has worsened your life situation? (select the corresponding figure)

 In a very high degree            not at all

 1          2         3         4         5

## References

[1] Allen PB, Gower-Rousseau C, Danese S, Peyrin-Biroulet L (2017). Preventing disability in inflammatory bowel disease. Therap Adv Gastroenterol.

[2] Gomollón F, Dignass A, Annese V (2017). 3rd European evidence-based consensus on the diagnosis and management of Crohn's disease 2016: part 1: diagnosis and medical management. J Crohns Colitis.

[3] Krauss E, Agaimy A, Gottfried A (2014). Long term follow up of through-the-scope balloon dilation as compared to strictureplasty and bowel resection of intestinal strictures in Crohn's disease. Int J Clin Exp Pathol.

[4] Abramson O, Durant M, Mow W (2010). Incidence, prevalence, and time trends of pediatric inflammatory bowel disease in Northern California, 1996 to 2006. J Pediatr.

[5] Torres J, Ellul P, Langhorst J (2019). European Crohn's and Colitis Organisation topical review on complementary medicine and psychotherapy in inflammatory bowel disease. J Crohns Colitis.

[6] National Center for Complementary and Integrative Health (NCCIH). Complementary a (2020). Complementary, alternative, or integrative health: what’s in a name?. https://www.nccih.nih.gov/health/complementary-alternative-or-integrative-health-whats-in-a-name.

[7] Hanssen B, Grimsgaard S, Launsø L, Fønnebø V, Falkenberg T, Rasmussen NK (2005). Use of complementary and alternative medicine in the Scandinavian countries. Scand J Prim Health Care.

[8] Harris PE, Cooper KL, Relton C, Thomas KJ (2012). Prevalence of complementary and alternative medicine (CAM) use by the general population: a systematic review and update. Int J Clin Pract.

[9] Opheim R, Hoivik ML, Solberg IC, Moum B; IBSEN Study Group (2012). Complementary and alternative medicine in patients with inflammatory bowel disease: the results of a population-based inception cohort study (IBSEN). J Crohns Colitis.

[10] Alrowais NA, Alyousefi NA (2017). The prevalence extent of complementary and alternative medicine (CAM) use among Saudis. Saudi Pharm J.

[11] Lönnfors S, Vermeire S, Greco M, Hommes D, Bell C, Avedano L (2014). IBD and health-related quality of life -- discovering the true impact. J Crohns Colitis.

[12] Harbord M, Eliakim R, Bettenworth D (2017). Third European evidence-based consensus on diagnosis and management of ulcerative colitis. Part 2: current management. J Crohns Colitis.

[13] Ordás I, Feagan BG, Sandborn WJ (2012). Therapeutic drug monitoring of tumor necrosis factor antagonists in inflammatory bowel disease. Clin Gastroenterol Hepatol.

[14] Bongartz T, Sutton AJ, Sweeting MJ, Buchan I, Matteson EL, Montori V (2006). Anti-TNF antibody therapy in rheumatoid arthritis and the risk of serious infections and malignancies: systematic review and meta-analysis of rare harmful effects in randomized controlled trials. JAMA.

[15] Bressler B, Law JK, Al Nahdi Sheraisher N (2008). The use of infliximab for treatment of hospitalized patients with acute severe ulcerative colitis. Can J Gastroenterol.

[16] Holleran G, Scaldaferri F, Gasbarrini A, Currò D (2020). Herbal medicinal products for inflammatory bowel disease: a focus on those assessed in double-blind randomised controlled trials. Phytother Res.

[17] Ng SC, Lam YT, Tsoi KK, Chan FK, Sung JJ, Wu JC (2013). Systematic review: the efficacy of herbal therapy in inflammatory bowel disease. Aliment Pharmacol Ther.

[18] Gröchenig HP, Waldhör T, Haas T (2019). Prevalence and indicators of use of complementary and alternative medicine in Austrian patients with inflammatory bowel disease. Eur J Gastroenterol Hepatol.

[19] van Tilburg MA, Palsson OS, Levy RL, Feld AD, Turner MJ, Drossman DA, Whitehead WE (2008). Complementary and alternative medicine use and cost in functional bowel disorders: a six month prospective study in a large HMO. BMC Complement Altern Med.

[20] Rawsthorne P, Shanahan F, Cronin NC, Anton PA, Löfberg R, Bohman L, Bernstein CN (1999). An international survey of the use and attitudes regarding alternative medicine by patients with inflammatory bowel disease. Am J Gastroenterol.

[21] Fernández A, Simian D, Quera R (2018). Complementary and alternative medicine in patients with inflammatory bowel disease: a survey performed in a tertiary center in Chile. Complement Ther Med.

[22] Chande N, Costello SP, Limketkai BN, Parker CE, Nguyen TM, Macdonald JK, Feagan BG (2020). Alternative and complementary approaches for the treatment of inflammatory bowel disease: evidence from Cochrane Reviews. Inflamm Bowel Dis.

[23] Hanai H, Iida T, Takeuchi K (2006). Curcumin maintenance therapy for ulcerative colitis: randomized, multicenter, double-blind, placebo-controlled trial. Clin Gastroenterol Hepatol.

[24] Lang A, Salomon N, Wu JC (2015). Curcumin in combination with mesalamine induces remission in patients with mild-to-moderate ulcerative colitis in a randomized controlled trial. Clin Gastroenterol Hepatol.

[25] Salomon N, Lang A, Gamus D (2015). Curcumin add-on therapy for ulcerative colitis. (Article in Hebrew). Harefuah.

[26] Koning M, Ailabouni R, Gearry RB, Frampton CM, Barclay ML (2013). Use and predictors of oral complementary and alternative medicine by patients with inflammatory bowel disease: a population-based, case-control study. Inflamm Bowel Dis.

[27] Lindberg A, Fossum B, Karlen P, Oxelmark L (2014). Experiences of complementary and alternative medicine in patients with inflammatory bowel disease - a qualitative study. BMC Complement Altern Med.

[28] Gallinger Z, Bressler B, Devlin SM, Plamondon S, Nguyen GC (2014). A survey of perceptions and practices of complementary alternative medicine among Canadian gastroenterologists. Can J Gastroenterol Hepatol.

[29] Cheifetz AS, Gianotti R, Luber R, Gibson PR (2017). Complementary and alternative medicines used by patients with inflammatory bowel diseases. Gastroenterology.

